# p38β MAPK mediates ULK1-dependent induction of autophagy in skeletal muscle of tumor-bearing mice

**DOI:** 10.15698/cst2018.11.163

**Published:** 2018-10-10

**Authors:** Zhelong Liu, Ka Wai Thomas Sin, Hui Ding, HoangAnh Amy Doan, Song Gao, Hongyu Miao, Yahui Wei, Yiman Wang, Guohua Zhang, Yi-Ping Li

**Affiliations:** 1 Department of Integrative Biology and Pharmacology, McGovern Medical School, University of Texas Health Science Center, Houston, Texas 77030, USA.; 2 Division of Endocrinology, Tongji Hospital, Tongji Medical College, Huazhong University of Science and Technology, Wuhan, China.; 3 Department of Respiratory Medicine, Yixing Hospital affiliated to Jiangsu University, Yixing, China.; 4 School of Public Health, University of Texas Health Science Center, Houston, Texas 77030, USA.

**Keywords:** cachexia, muscle wasting, C/EBPβ, LC3b, Gabarapl1, ULK1

## Abstract

Muscle wasting is the key manifestation of cancer-associated cachexia, a lethal metabolic disorder seen in over 50% of cancer patients. Autophagy is activated in cachectic muscle of cancer hosts along with the ubiquitin-proteasome pathway (UPP), contributing to accelerated protein degradation and muscle wasting. However, established signaling mechanism that activates autophagy in response to fasting or denervation does not seem to mediate cancer-provoked autophagy in skeletal myocytes. Here, we show that p38β MAPK mediates autophagy activation in cachectic muscle of tumor-bearing mice via novel mechanisms. Complementary genetic and pharmacological manipulations reveal that activation of p38β MAPK, but not p38α MAPK, is necessary and sufficient for Lewis lung carcinoma (LLC)-induced autophagy activation in skeletal muscle cells. Particularly, muscle-specific knockout of p38β MAPK abrogates LLC tumor-induced activation of autophagy and UPP, sparing tumor-bearing mice from muscle wasting. Mechanistically, p38β MAPK-mediated activation of transcription factor C/EBPβ is required for LLC-induced autophagy activation, and upregulation of autophagy-related genes LC3b and Gabarapl1. Surprisingly, ULK1 activation (phosphorylation at S555) by cancer requires p38β MAPK, rather than AMPK. Activated ULK1 forms a complex with p38β MAPK in myocytes, which is markedly increased by a tumor burden. Overexpression of a constitutively active p38Tbeta; MAPK in HEK293 cells increases phosphorylation at S555 and other amino acid residues of ULK1, but not several of AMPK-mediated sites. Finally, ULK1 activation is abrogated in tumor-bearing mice with muscle-specific knockout of p38β MAPK. Thus, p38β MAPK appears a key mediator of cancer-provoked autophagy activation, and a therapeutic target of cancer-induced muscle wasting.

## INTRODUCTION

Cancer has been increasingly recognized as a systemic disorder that stresses multiple organs independent of its location. At least 50% of cancer patients experience cachexia, a systemic wasting syndrome manifested as weight loss, inflammation, insulin resistance, and increased muscle protein breakdown. Progressive loss of muscle mass (muscle wasting) contributes significantly to cancer-associated morbidity and mortality 1,2. However, the etiology of cancer cachexia is not well defined and there is no FDA-approved treatment for this lethal disorder.

There has been a general consensus that accelerated muscle protein degradation is a major cause of cachexia-associated muscle mass loss. It has been well-established that the ubiquitin proteasome pathway (UPP) plays an important role in cancer-induced muscle wasting by degrading myofibrillar proteins 3,4,5. More recent evidence indicates that cancer also induces autophagy activation in the cachectic muscle of tumor-bearing mice 6,7,8,9 and cancer patients 10,11,12. Autophagy targets cytoplasmic constituents including ubiquitinated protein aggregates and organelles for degradation by lysosomes 13,14. Autophagy inhibition blocks muscle protein degradation induced by the activation of Toll-like receptor 4 (TLR4) 15, a plasma membrane receptor that is activated by danger-associated molecular patterns (DAMPs)^16^ and mediates cancer-induced muscle wasting 4,9,17. Thus, cancer-provoked autophagy activation is considered a therapeutic target of cancer-induced muscle wasting. However, the intramuscular signaling pathways that mediate cancer-induced activation of autophagy remain poorly understood. Elucidating and thereby targeting the cellular signaling pathways that mediate cancer-induced activation of autophagy could allow intervention of cancer-induced muscle wasting.

The Akt-FoxO signaling pathway inversely mediates the activity of both autophagy and UPP in muscle in response to such catabolic stimuli as fasting or denervation 18,19. However, Akt is activated in cachectic muscle of tumor-bearing mice 20,21 and cancer patients 11,12, which inhibits FoxOs by promoting their translocation out of nuclei 22. Thus, the Akt-FoxO signaling pathway does not appear to mediate cancer-induced muscle catabolism that is due largely to systemic inflammation. On the other hand, we observed previously that inflammation activated-p38 MAPK mediates both the autophagy and UPP activation in skeletal muscle in response to TLR4 activation by lipopolysaccharide 15. Other inflammatory mediators implicated in cancer cachexia also activate p38 MAPK including oxidative stress 23, TNFα 24, IL-6 25, IL-1 26, TWEAK 27, activin A/myostatin 28 as well as extracellular Hsp70 and Hsp90 4, while promoting autophagy and/or UPP-mediated muscle protein loss. However, it is unknown which of the three p38 MAPK isoforms that are expressed in skeletal muscle ((, (, and () mediates cancer-induced autophagy activation. More importantly, how p38 MAPK activates autophagy is unknown.

In the present study, we demonstrate that activation of the p38β MAPK isoform is necessary and sufficient for autophagy activation in skeletal muscle in a mouse model of cancer cachexia, and that deletion of p38β MAPK in skeletal muscle abrogates muscle wasting by attenuating muscle protein degradation mediated by autophagy as well as UPP. Mechanistically, p38Tbeta; MAPK mediates cancer-provoked autophagy activation by upregulating Atg8 orthologues LC3b and Gabarapl1 as well as by activating ULK1. These data support p38β MAPK as a key mediator and therapeutic target of cancer-associated muscle wasting.

## RESULTS 

### LLC induces autophagy activation in skeletal muscle cells through p38β MAPK

**Figure 1:**
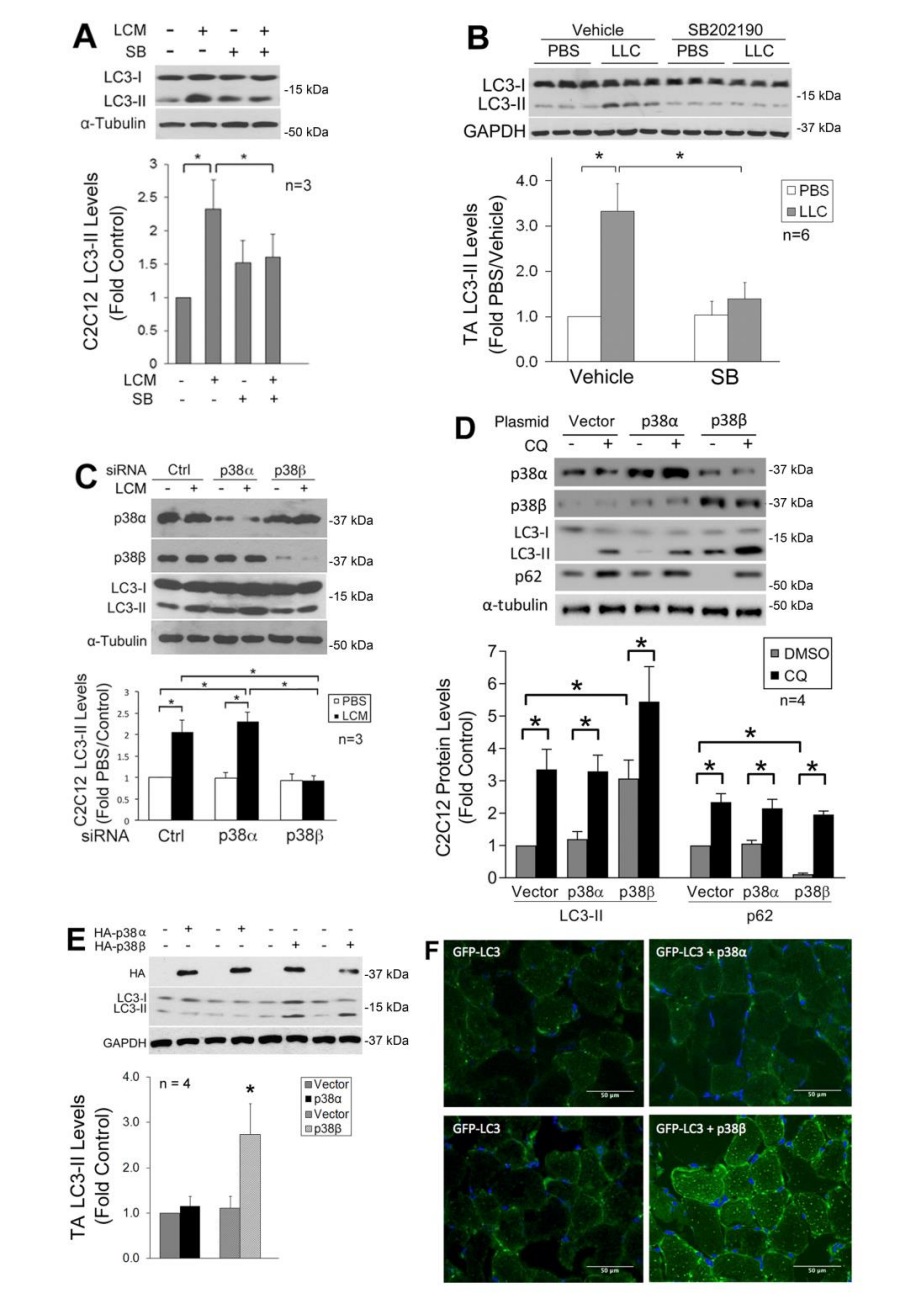
FIGURE 1: p38β MAPK activation is necessary and sufficient for autophagy activation by tumor in skeletal muscle cells. **(A)** Inhibition of p38 MAPK attenuates LLC-conditioned medium (LCM)-induced autophagy activation in C2C12 myotubes. C2C12 myotubes were pretreated with SB202190 (SB, 10 (M) or vehicle (0.1% DMSO) for 30 min prior to treatment with LCM or control medium (NL20) for 8 h. Autophagy activation was evaluated by Western blotting analysis of LC3 in cell lysate. **(B)** Inhibition of p38 MAPK attenuates autophagy activation in skeletal muscle of LLC tumor-bearing mice. In 7 days of LLC implant to mice, SB202190 was i.p. injected (5 mg/kg) daily with DMSO (50%) as vehicle control for 14 days. Lysate of TA collected on 21 days of LLC implant was analyzed by Western blotting for LC3 (samples from 3 mice per group were loaded on each gel). **(C) **LCM-induced autophagy activation in myotubes is dependent on p38β MAPK. C2C12 myoblasts were transfected with control, p38α MAPK or p38β MAPK-specific siRNA. After differentiation, myotubes were treated with LCM or control medium for 8 h. Autophagy activation was evaluated by Western blotting analysis of LC3. **(D)** Expression of constitutively active p38β MAPK stimulates autophagy flux in C2C12 myotubes. Plasmids encoding a constitutively active mutant of p38α MAPK or p38β MAPK with the HA tag were transfected into C2C12 myoblasts, with empty vector as control. After differentiation, myotubes were treated with chloroquine (CQ, 20 (M) for 8 h. LC3, p62 and expression of the p38 MAPK mutants were monitored by Western blotting. **(E)** Constitutively active p38β MAPK activates autophagy in mouse muscle. Plasmids encoding the constitutively active mutant of p38α MAPK or p38β MAPK fused with the HA tag were transfected into the TA of mice. Empty vector was transfected into the contralateral TA. On day 14 expression of the p38 MAPK mutants and autophagy activation was analyzed by Western blotting (data from two mice per group are shown). Data were analyzed by one way ANOVA. * denotes a difference between bracketed groups or from controls (p < 0.05). **(F)** Constitutively active p38β MAPK stimulates autophagosome formation in mouse muscle. A plasmid encoding GFP-LC3 was transfected into mouse TA muscle, and co-transfected with the plasmid encoding the constitutively active mutant of p38α MAPK or p38β MAPK in the contralateral TA. In 7 days, TA muscle was collected. Frozen sections were prepared and stained with DAPI. Autophagosome formation was evaluated by confocal microscopy. Bars represent 50 μm.

We previously showed that LLC induces an increase in autophagy flux and autophagosome formation in cultured C2C12 myotubes as well as mouse muscle 9. In the present study, we further investigated whether LLC induces autophagy activation in skeletal muscle cells through p38 MAPK by monitoring the autophagy marker LC3-II. By pretreating C2C12 myotubes, which respond to cancer cell-conditioned media in a similar manner as primary myotubes 4, with a p38α/β MAPK inhibitor SB202190, we observed that the induction of autophagy by LLC cell-conditioned medium (LCM) required p38 MAPK activation (**Figure 1A**). In addition, systemic administration of SB202190 to LLC tumor-bearing mice inhibited autophagy activation in cachectic muscle (**Figure 1B**). These results suggest that LLC induces autophagy activation in skeletal muscle through the activation of p38α and/or p38β MAPK.

To identify the isoform of p38 MAPK that mediated LLC induction of autophagy, we utilized siRNA-mediated gene silencing and observed that the loss of p38β MAPK, but not p38α MAPK, abolished LCM-induced autophagy activation (**Figure 1C**). To examine whether activated p38β MAPK actually stimulated autophagy flux, a constitutively active mutant of p38α MAPK or p38β MAPK 29 was expressed in myotubes. Only the active p38β MAPK increased the LC3-II level. In addition, treatment of myotubes with lysosome inhibitor chloroquine (CQ) further increased LC3-II in active p38β MAPK-expressing myotubes. Further, expression of active p38β MAPK resulted in a loss in the autophagy-selective target p62, and CQ treatment abrogated this effect (**Figure 1D**). These results indicate that p38β MAPK activation stimulated autophagy flux. Similarly, LC3-II was specifically increased by the overexpressed active mutant of p38β MAPK in the muscle (TA) of tumor-free mice (**Figure 1E**). Furthermore, co-transfection of GFP-LC3 with active p38β MAPK, but not active p38α MAPK, in mouse muscle resulted in increased autophagosome formation as indicated by the formation of GFP-LC3-incorporated puncta (**Figure 1F**). These data provided evidence that p38β MAPK activation is necessary and sufficient for autophagy activation in skeletal muscle cells by a tumor burden.

### LLC induces muscle wasting through p38β MAPK-mediated activation of autophagy and UPP

**Figure 2:**
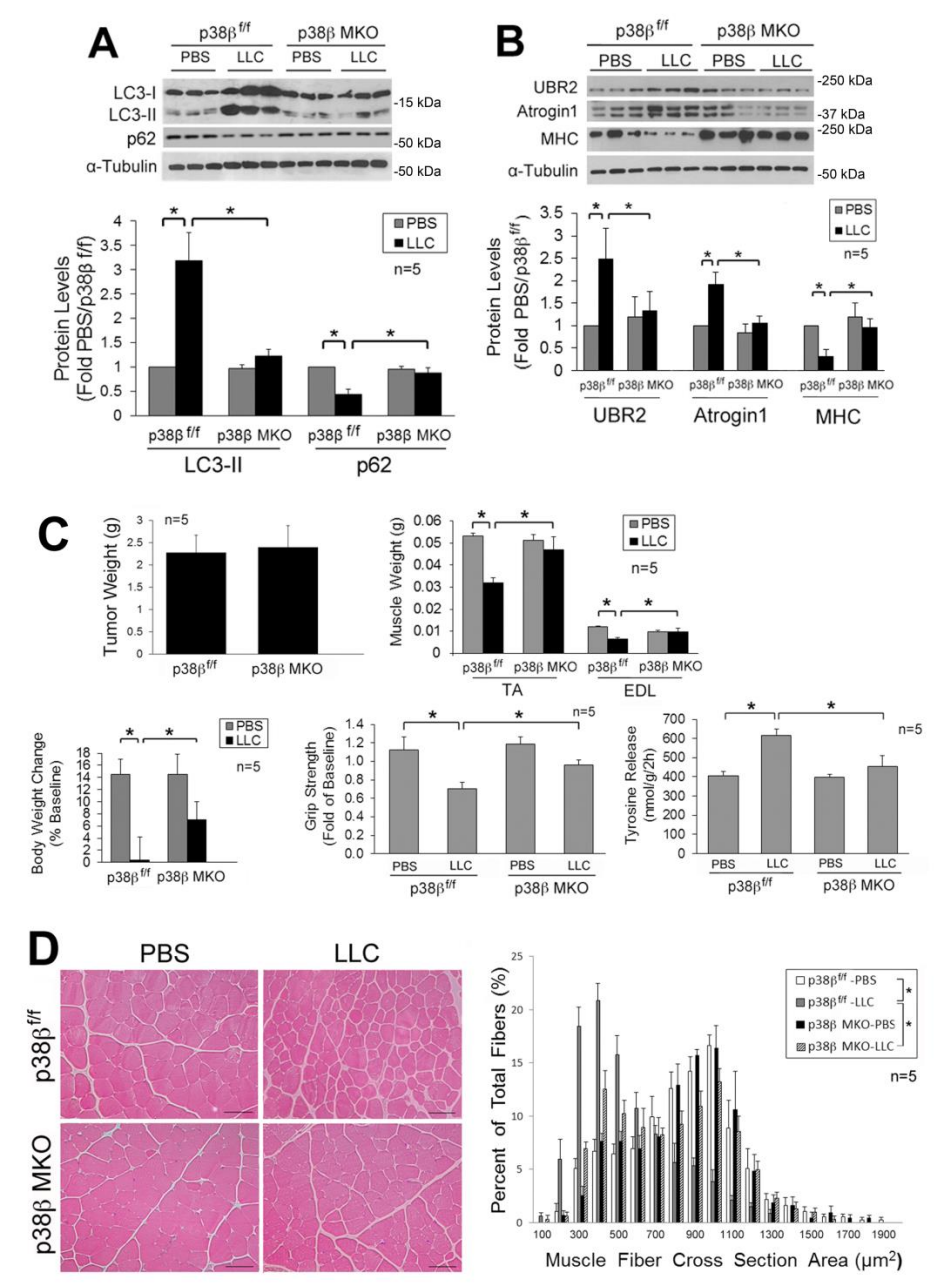
FIGURE 2: Muscle-specific knockout of p38β MAPK abrogates activation of autophagy, UPP and muscle wasting in tumor-bearing mice. Mice with muscle-specific knockout of p38β MAPK and control mice (p38β MAPK^f/f^) were implanted with LLC cells. In 21 days, mice were euthanized for analysis of muscle wasting. Lysate of TA collected from the mice was analyzed by Western blotting for LC3 and p62 to evaluate autophagy activity **(A) **and UBR2, atrogin1 and MHC to evaluate UPP activity **(B)**. Muscle wasting in the mice was analyzed by examining tumor weight, body and muscle weight, tyrosine release and grip strength **(C)**. Muscle mass was measured as muscle fiber cross sectional area **(D)**. Data in A to C were analyzed by one way ANOVA. Data in D were analyzed by Chi-square analysis. * denotes a difference (p < 0.05).

Next, we investigated whether LLC-induced activation of autophagy and muscle wasting are attenuated in the muscle of mice with muscle-specific knockout of p38β MAPK (p38β MKO) 30. It was previously shown that the muscle phenotype was not altered by deleting the *p38*β gene 28,30,31. We observed that p38β MKO mice were resistant to LLC-induced autophagy activation as measured by LC3-II and p62 levels (**Figure 2A**). In addition, p38β MKO mice were resistant to LLC-stimulated UPP activity as monitored by the levels of atrogin1, UBR2 and myofibrillar protein myosin heavy chain (MHC) (**Figure 2B**). Consequently, p38β MKO mice were spared from LLC-induced muscle wasting without altering tumor growth as measured by tumor weight, body and muscle weight, muscle proteolysis (tyrosine release), muscle strength (grip strength, **Figure 2C**) and myofiber cross-sectional area (**Figure 2D**). Thus, LLC induces muscle wasting through p38β MAPK-mediated activation of both autophagy and UPP.

### p38β MAPK-mediated C/EBPβ activation is critical to LLC-induced autophagy activation

**Figure 3:**
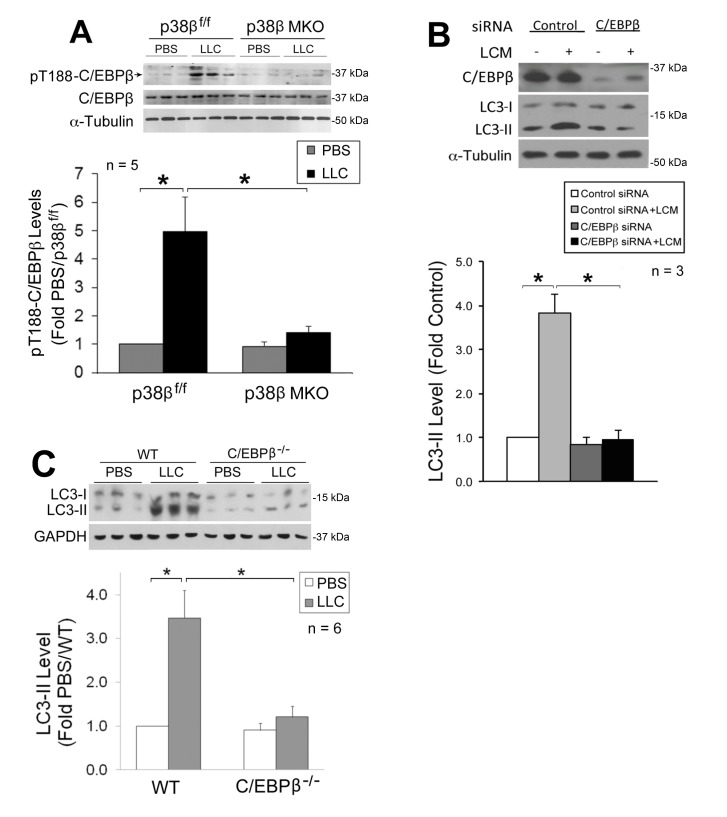
FIGURE 3: The p38β MAPK → C/EBPβ signaling pathway mediates autophagy activation by tumor in skeletal muscle cells. **(A)** C/EBPβ activation in skeletal muscle of LLC tumor-bearing mice is dependent on p38β MAPK. Lysate of TA collected from p38β MAPK^f/f^ and p38β MAPK muscle-specific knockout mice on day 21 of LLC implant was analyzed by Western blotting for C/EBPβ activation (phosphorylation of Thr-188). **(B)** C/EBPβ is critical to LCM-induced autophagy activation in myotubes. C2C12 myoblasts transfected with control or C/EBPβ-specific siRNA were treated with LCM or control medium for 8 h. C/EBPβ and LC3 were analyzed by Western blotting. **(C)** Autophagy activation in skeletal muscle of LLC tumor-bearing mice is dependent on C/EBPβ. Lysate of TA collected from wild type and C/EBPβ knockout mice on day 21 of LLC implant was analyzed by Western blotting for LC3.

We previously showed that p38β MAPK mediates LLC-induced upregulation of atrogin1 29 and UBR2 32 through the activation of C/EBPβ-binding to a cis-element in their 5’-promoter by phosphorylating the Thr-188 residue of C/EBPβ 29, and that LLC-induced muscle wasting is abrogated in C/EBPβ knockout mice 21. We therefore investigated whether the p38β MAPK → C/EBPβ signaling pathway also mediates LLC activation of autophagy. We observed that LLC-induced activation of C/EBPβ was blocked in the muscle of p38β MKO mice (**Figure 3A**), confirming *in vivo* that p38β MAPK mediates LLC-induced activation of C/EBPβ. To investigate whether C/EBPβ is critical to LLC-induced autophagy activation, we treated C/EBPβ-deficient C2C12 myotubes with LCM, and observed a dependence of LCM-induced autophagy activation on C/EBPβ (**Figure 3B**). Further, we observed that LLC-induced autophagy activation was abrogated in the muscle of C/EBPβ knockout mice (**Figure 3C**). Thus, p38β MAPK-mediated activation of C/EBPβ is critical to LLC-induced autophagy activation.

### p38β MAPK → C/EBPβ signaling mediates upregulation of specific autophagy-related genes 

**Figure 4:**
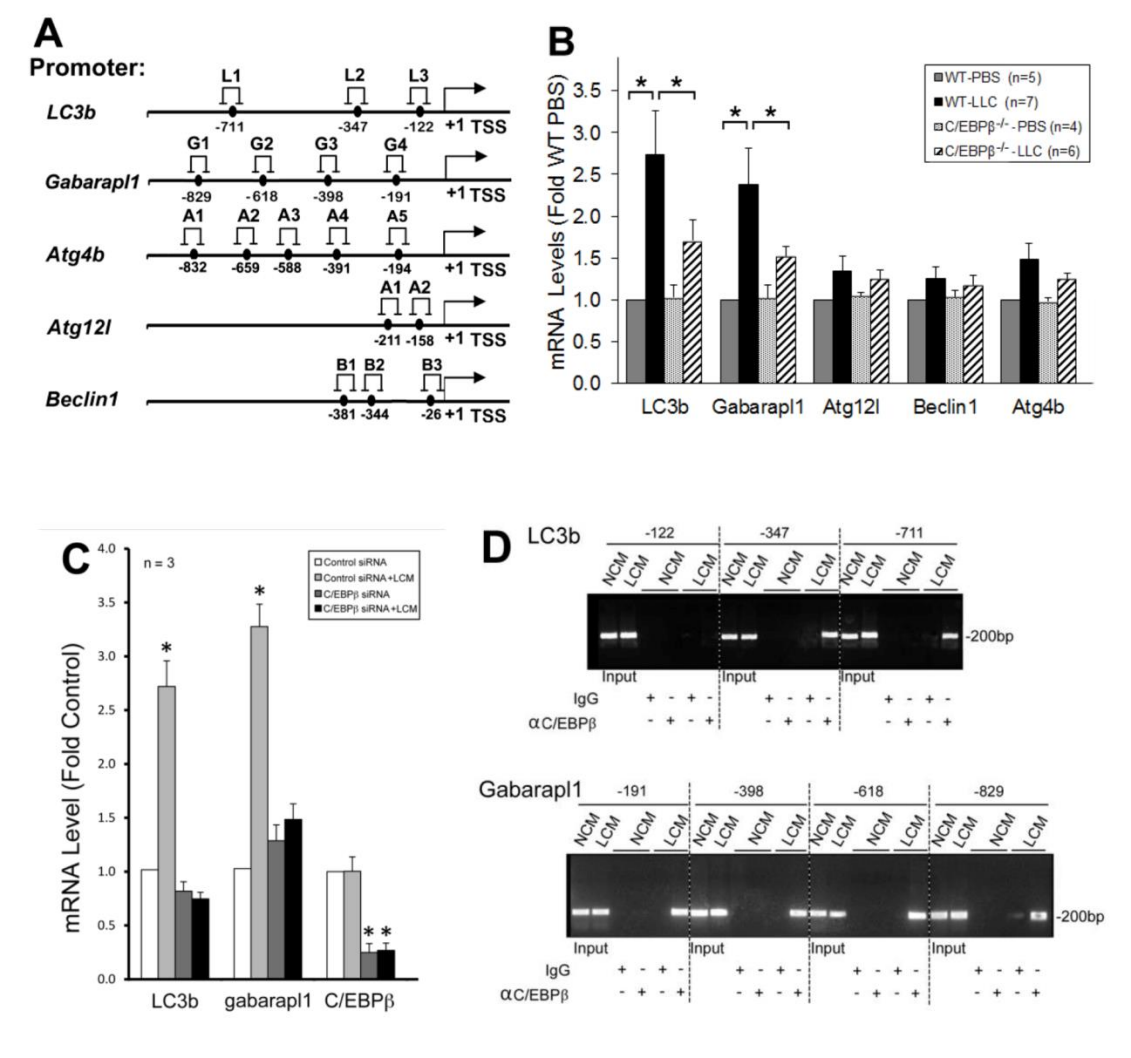
FIGURE 4. C/EBPβ mediates tumor induction of autophagy-related genes in skeletal muscle cells. **(A)** Potential C/EBPβ-binding cites are identified in autophagy-related genes. Data base search identified multiple potential biding sites of C/EBPβ in the 5’ promoter region of listed autophagy-related genes. **(B)** *LC3b* and *Gabarapl1* are upregulated in skeletal muscle of LLC tumor-bearing mice in a C/EBPβ-dependent manner. Total RNA was extracted from the TA of wild type and C/EBPβ knockout mice implanted with LLC cells for 21 days, and analyzed for the mRNA of above identified autophagy-related genes by real-time PCR. **(C)** *LC3b* and *Gabarapl1* are upregulated in LCM-treated myotubes in a C/EBPβ-dependent manner. Total RNA was extracted from C2C12 myotubes transfected with C/EBPβ-specific or control siRNA. The mRNA of *C/EBPβ*, *LC3b* and *Gabarapl1* was determined by real-time PCR. Data from panel B and C were analyzed by one way ANOVA. * denotes a difference (p < 0.05). **(D)** LCM activates C/EBPβ binding to the *LC3b* and *Gabarapl1* promoters in myotubes. C/EBPβ binding to the *LC3b* and *Gabarapl1* promoters in C2C12 myotubes treated with LCM or the control NL20 cell-conditioned medium (NCM) was analyzed by the ChIP assay. Pre-immune IgG was used as control for C/EBPβ-specific antibody.

To understand how C/EBPβ mediates LLC-induced autophagy activation, we searched a data base (http://tfbind.hgc.jpfor potential C/EBPβ-binding sites in the 5’-promoter regions of autophagy-related genes and identified multiple sites within -1 kilo-bases in five important autophagy related genes (**Figure 4A**). By analyzing mRNA levels of these genes in the muscle of LLC tumor-bearing wild type and C/EBPβ knockout mice, we found that mRNA of *LC3b and Gabarapl1, *two *Atg8* orthologues*, *were upregulated by LLC in a C/EBPβ-dependent manner (**Figure 4B**)*. *Corroborative data were obtained in C2C12 myotubes where LCM treatment upregulated the two genes in a C/EBPβ-dependent manner (**Figure 4C**). To determine whether LLC stimulates C/EBPβ binding to the two gene promoters, we performed the chromatin immunoprecipitation (ChIP) assay and observed that LCM treatment of C2C12 myotubes stimulated C/EBPβ binding to multiple sites in the *LC3b and Gabarapl1 *promoter (**Figure 4D**). These data suggest that C/EBPβ upregulates the two *Atg8* orthologues in muscle cells in response to a tumor burden.

**Figure 5:**
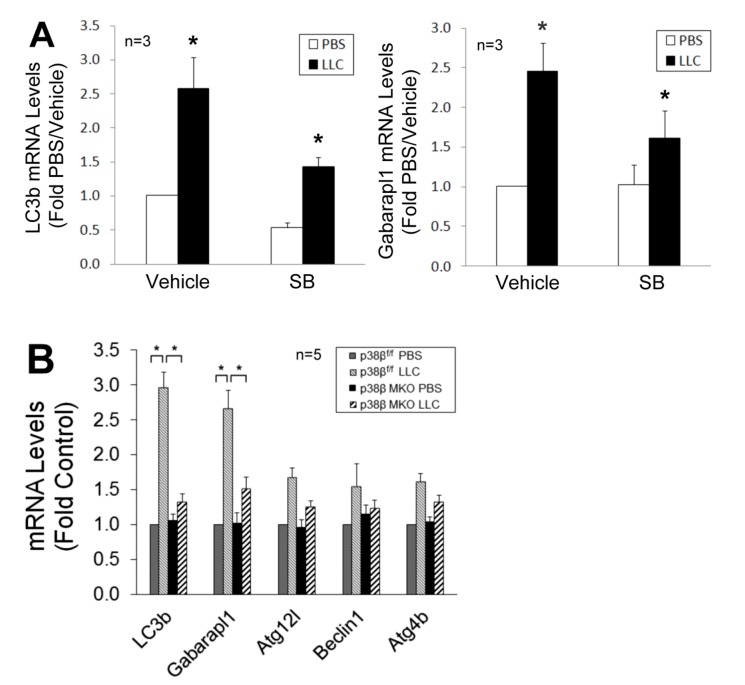
FIGURE 5: p38β MAPK is required for upregulation of C/EBPβ-responsive autophagy-related genes in cachectic muscle of tumor-bearing mice. **(A)** Inhibition of p38 MAPK attenuates *LC3b* and *Gabarapl1* upregulation in cachectic muscle of LLC tumor-bearing mice. On day 7 of LLC cells implant to mice, SB202190 was i.p. injected (5 mg/kg) daily with DMSO (50%) as vehicle control. Lysate of TA collected on day 21 was analyzed by qPCR for *LC3b* and *Gabarapl1 *mRNA levels. **(B)** *LC3b* and *Gabarapl1* upregulation in the muscle of LLC tumor-bearing mice is dependent on p38β MAPK. Mice with muscle-specific knockout of p38β MAPK (p38β MAPK MKO) or control mice (p38β MAPK^f/f^) were implanted with LLC cells. On day 21, total RNA was extracted from TA of the mice and analyzed by qPCR for *LC3b* and *Gabarapl1 *mRNA levels. Data were analyzed by one way ANOVA. * denotes a difference (p < 0.05).

To verify whether p38β MAPK is required for C/EBPβ-mediated upregulation of *LC3b *and* Gabarapl1*, we observed that the upregulation of these genes in LCM-treated myotubes was inhibited by SB202190 (**Figure 5A**). Further, upregulation of these genes in skeletal muscle was abrogated in LLC tumor*-*bearing p38β MKO mice (**Figure 5B**). These data confirm that LLC upregulates *LC3b *and *Gabarapl1 *through the p38β MAPK ( C/EBPβ signaling pathway.

### LLC induces ULK1 activation in skeletal muscle through p38β MAPK 

**Figure 6:**
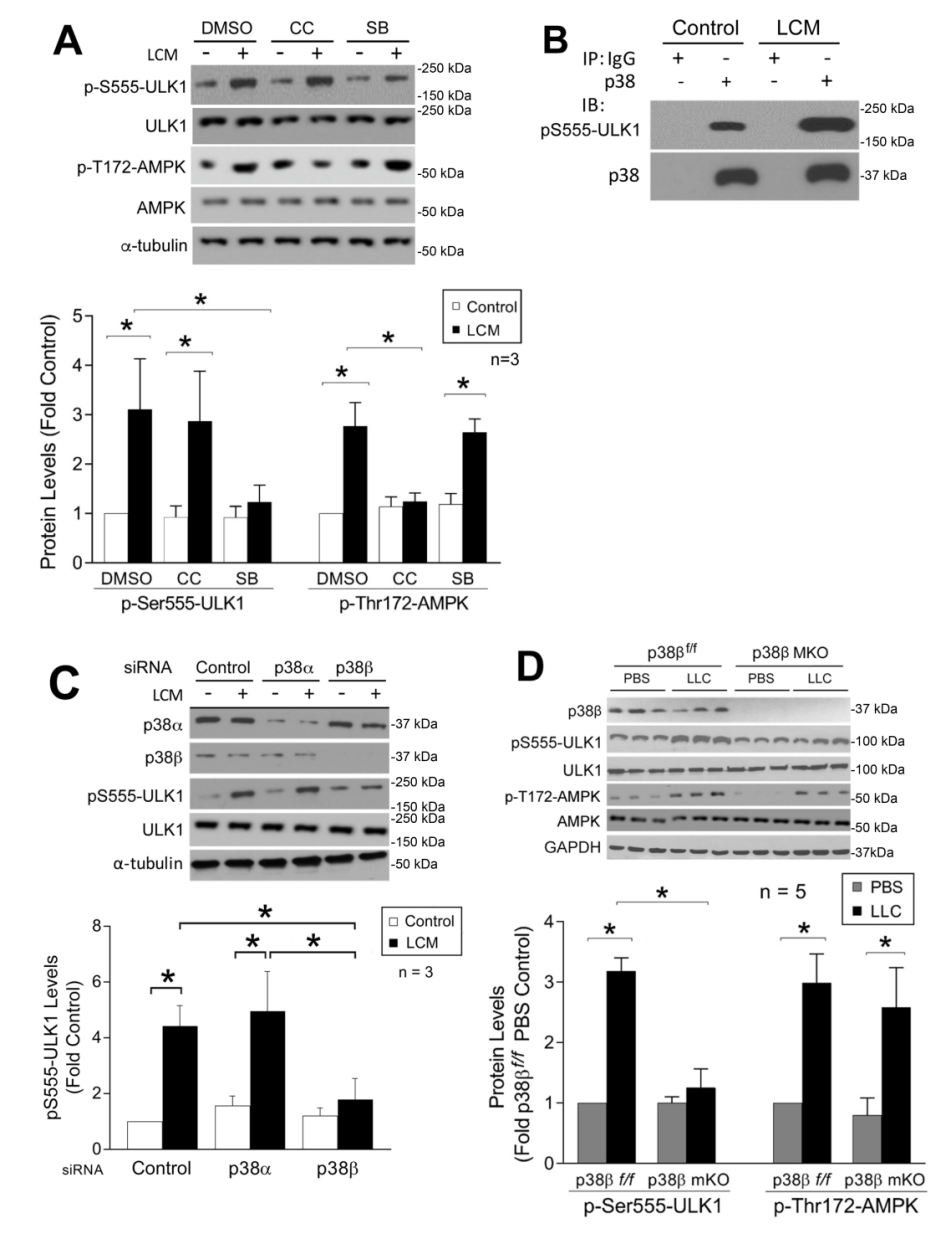
FIGURE 6: p38β MAPK activates ULK1 in skeletal muscle cells in response to tumor. **(A)** LCM stimulates ULK1 phosphorylation on Ser-555 in myotubes through p38 MAPK. C2C12 myotubes were pretreated with DMSO (0.1%, vehicle), compound C (CC, 10 (M) or SB202190 (SB, 10 (M) for 30 min, and then treated with LCM for 1 h. ULK1 phosphorylation state on Ser-555 and AMPK phosphorylation on Thr-172 in cell lysate were analyzed by Western blotting. **(B)** LCM stimulates p38 MAPK interaction with ULK1 in myotubes. C2C12 myotubes were treated with LCM for 1 h. Cell lysate was subjected to immunoprecipitation with pre-immune IgG or p38 MAPK-specific antibody. Precipitate was analyzed by Western blotting for p38 MAPK and pSer-555 ULK1. **(C)** LCM stimulation of ULK1 phosphorylation on Ser-555 in myotubes is mediated by p38β MAPK specifically. C2C12 myoblasts were transfected with control, p38β MAPK or p38β MAPK-specific siRNA. After differentiation for 96 h, myotubes were treated with LCM or control medium for 1 h. Knockdown of p38 MAPKs and phosphorylation of ULK1 on Ser-555 were analyzed by Western blotting. **(D)** p38β MAPK is required for ULK1 activation in cachectic muscle of LLC tumor-bearing mice. Mice with muscle-specific knockout of p38β MAPK and control mice were implanted with LLC cells. Lysate of TA collected from these mice on day 21 was analyzed by Western blotting for ULK1 phosphorylation on Ser-555 and AMPK phosphorylation on Thr-172. Data were analyzed by one way AVOVA. * denotes a difference (p < 0.05).

Both LC3b and Gabarapl1 are members of the ATG8 family that are essential for autophagosome formation 33. On the other hand, ATG8 family members must be activated by the ULK1 complex post-translationally to initiate the lipidation process required for autophagsome formation 34. Therefore, we examined whether LLC induced ULK1 activation in skeletal muscle cells. LCM treatment of C2C12 myotubes for 1 h increased ULK1 phosphorylation on Ser-555 (pS555-ULK1, **Figure 6A**). Phosphorylation of ULK1 on this serine residue is known to activate ULK1 by AMP-activated protein kinase (AMPK) upon nutrient deprivation 35,36. LCM was shown previously to induce mTOR inhibition and AMPK activation in C2C12 myotubes 37. To examine the involvement of AMPK in LCM-induced activation of ULK1, we pretreated myotubes with the AMPK inhibitor Compound C 38. To our surprise, Compound C did not alter LCM-induced ULK1 phosphorylation on Ser-555, although it did inhibit AMPK activation as measured by the phosphorylation state of its Thr-172 residue. However, pretreatment with p38 MAPK inhibitor SB202190 attenuated LCM-induced Ser-555 phosphorylation of ULK-1, without affecting LCM-induced Thr-172 phosphorylation of AMPK (**Figure 6A**). These data suggest that p38 MAPK, rather than AMPK, is critical to LCM-induced activation of ULK1. We then investigated whether p38 MAPK interacted with ULK1 by performing immunoprecipitation of p38 MAPK from myotube lysate. We observed co-precipitation of pS555-ULK1 with p38 MAPK in control myotubes, and that the level of co-precipitated pS555-ULK1 dramatically increased in LCM-treated myotubes (**Figure 6B**), suggesting that p38 MAPK does interact with ULK1 resulting in phosphorylation of its Ser-555 residue in myocytes, and this activity is stimulated upon p38 MAPK activation by a tumor burden. To identify the p38 MAPK isoform responsible for LCM-stimulated ULK1 phosphorylation on Ser-555, we performed siRNA-mediated gene silencing in myotubes and observed that p38β MAPK, but not p38α MAPK, was required for this reaction (**Figure 6C**). To verify that activation of p38β MAPK is sufficient to phosphorylate ULK1, FLAG-tagged ULK1 was co-expressed with constitutively active p38β MAPK in HEK293T cells. Mass spectrometry analysis of FLAG-ULK1 isolated from cell lysate revealed multiple phosphorylated amino acid residues including Ser-555 (Supplementary Table 1). However, these sites did not include a number of known AMPK-mediated phosphorylation sites in ULK1 such as Ser-317, Ser-467, Ser-637 and Ser-777 36,39. In combination, the above data support that p38β MAPK stimulates ULK1 activity upon activation by cancer independent of AMPK in the cellular environment. Finally, we confirmed that Ser-555 phosphorylation of ULK1 increased in the muscle of LLC tumor-bearing p38β^f/f^ mice but not in that of muscle-specific p38β MAPK knockout mice. On the other hand, LLC-induced AMPK activation was not altered in p38β MAPK-deficient muscle (**Figure 6E**). Therefore, we conclude that p38β MAPK, rather than AMPK, mediates LLC-induced autophagosome formation by activating ULK1 in addition to upregulating LC3b and Gabarapl1.

## DISCUSSION

The current study identifies p38β MAPK as a key mediator of cancer-induced autophagy activation in skeletal muscle, through activating ULK1 as well as upregulating C/EBPβ-controlled *LC3b* and *Gabarapl1* genes. Importantly, we were able to abrogate cancer-induced muscle wasting by deleting p38β MAPK in skeletal muscle to prevent muscle protein degradation mediated by autophagy as well as UPP. These findings suggest that p38β MAPK is a key mediator and a therapeutic target of cancer-induced muscle wasting.

We show that p38β MAPK upregulates *LC3b* and *Gabarapl1* through activating C/EBPβ. The LC3 and Gabarapl family of proteins are mammalian orthologues of ATG8, a yeast autophagy-related protein involved in the formation of autophagosomes 33. Increased expression of LC3/Gabaraple and activation of autophagy have been observed in cachectic muscle of cancer patients 10,11,12. In various types of cells, increased expression of LC3/Gabarapl are often associated with autophagy activation 40,41. As the substrates of ULK1-mediated lipidation process that is critical for autophagosome formation 34, increased expression of LC3/Gabarapl is likely to facilitate autophagosome formation. On the other hand, the possibility exists that C/EBPTbeta; may regulate additional autophagy-related genes that are rate-limiting for autophagosome formation.

Autophagy is activated by nutritional deprivation through AMPK-mediated phosphorylation of ULK1 on multiple sites including Ser-555 in various cell types 35,36. In skeletal muscle, autophagy is also activated by the inactivation of Akt that inversely regulates FoxO3-mediated upregulation of autophagy-related gene expression in response to denervation or starvation 18,42, or by AMPK-mediated activation of FoxO3 in response to C26 colon adenocarcinoma 43. We previously showed that LLC activates Akt and inactivates FoxO1/3 in muscle cells, while activating p38 MAPK concomitantly 21. In the present study we show that despite AMPK activation by LLC in skeletal muscle, AMPK does not mediate in ULK1 activation LLC tumor-bearing mice. On the contrary, autophagy activation in skeletal muscle by LLC requires p38β MAPK-mediated activation of ULK1. Thus, tumor activates autophagy in skeletal muscle through a signaling mechanism distinct from that in nutritional deprivation.

It is well-established that in skeletal muscle p38 MAPK is activated by various inflammatory mediators implicated in cancer cachexia including various cytokines 24,25,26,27,28, reactive oxygen species (ROS)^23^ and extracellular vesicle-associated Hsp70 and Hsp90 4. Of the three members of p38 MAPK family expressed in skeletal muscle, p38α MAPK is responsible for most of the known roles of p38 MAPK including mediating inflammation 44,45 and promoting myogenesis 46,47. p38γ MAPK regulates the expansion of myogenic precursor cells 48, endurance exercise-induced mitochondrial biogenesis and angiogenesis 30, as well as glucose uptake 49. On the other hand, p38β MAPK has few known functions. Utilizing genetic manipulations including the p38β MAPK muscle specific knockout mice 30 in addition to pharmacological inhibition of p38α/β MAPK (due to the lack of p38β MAPK-specific inhibitors), the current study demonstrated for the first time that p38β MAPK is essential to autophagy activation during muscle wasting induced by LLC. The current study also demonstrated for the first time *in vivo* that p38β MAPK is essential to UPP activation and muscle wasting induced by cancer. Therefore, developing p38β MAPK-specific pharmacological inhibitors would be highly desirable for the intervention of cancer cachexia.

Our findings on the role of p38β MAPK in autophagy activation may have significance beyond skeletal muscle cells. For example, p38 MAPK mediates various stress-induced autophagy activation in a variety of cells 50,51,52,53,54. Particularly, in response to TLR4 activation, p38 MAPK mediates autophagy activation associated with innate immunity 55, which is similar to cancer-induced muscle wasting 4,9,17. However, which p38 MAPK isoform and how it activates autophagy in those processes are unknown. It is possible that a similar mechanism as we observed in skeletal muscle cells exists in other types of cells to mediate autophagy activation in response to inflammatory stresses.

Taken together, our findings reveal that p38β MAPK mediates cancer-induced autophagy in skeletal muscle through novel transcriptional as well as post-translational mechanisms. In addition, our findings suggest that cancer-induced muscle wasting can be ameliorated by targeting a single signaling molecule in skeletal muscle, p38β MAPK.

## MATERIALS AND METHODS

### Myogenic cell culture

Murine C2C12 myoblasts (American Type Culture Collection) were cultured in growth medium (DMEM supplemented with 10% fetal bovine serum) at 37(C with 5% CO_2_. At 85-90% confluence, myoblast differentiation was induced by incubation for 96 h in differentiation medium (DMEM supplemented with 4% heat-inactivated horse serum) to form myotubes. Preconditioned medium from cultures of Lewis lung carcinoma cells (obtained from National Institute of Cancer, Frederick, MD) or non-tumorigenic human lung epithelial cell line NL20 (obtained from American Type Culture Collection) that were cultured for 48 h were centrifuged and the supernatant was used to treat C2C12 myotubes (25% final volume in fresh medium) when indicated. Pretreatment of SB202190 or compound C (10 μM, dissolved in DMSO with 0.1% final concentration, Sigma-Aldrich, St. Louis, MO) for 30 min was carried out when indicated. All cell culture experiments were independently replicated 3 times as indicated (N = 3).

### Animal use

Experimental protocols were approved in advance by the institutional Animal Welfare Committee at the University of Texas Health Science Center at Houston. For LLC cell xenograft, 100 (l LLC (National Institute of Cancer) cells (1 × 10^6 ^cells), or an equal volume of vehicle (PBS) was injected subcutaneously into the right flanks of 7-week-old male C57BL/6 mice (The Jackson Laboratory, Bar Harbor, ME), C/EBPβ^-/-^ mice in C57BL/6 background that were bred from C/EBPβ^-/+ ^mice 56 or mice with muscle-specific knockout of p38β (p38β MKO) 28. The latter were created by crossbreeding floxed-p38β mice (p38β^ f/f^) in C57BL/6 background 30 with muscle creatine kinase-Cre (MCK-Cre, The Jackson Laboratory, Bar Harbor, ME). SB202190 was i.p. injected (5 mg/kg) daily from day 7 of tumor implant. Mice were euthanized on day 21 for evaluation of muscle wasting. When indicated plasmids encoding constitutively active mutant of p38 MAPK isoforms and GFP-LC3 fusion protein 55 were transfected into TA by electroporation as previously described 29.

### Transfection of siRNA and plasmids in C2C12 myoblasts

Predesigned siRNAs specific for C/EBPβ, p38α and p38β were purchased form Sigma-Aldrich. The IDs of the siRNAs were SASI_Mm01_00187563, SASI_Mm01_00020743 and SASI_Mm01_00044863, respectively. Control siRNA was purchased from Invitrogen. These siRNAs were introduced into C2C12 myoblasts using the jetPRIME reagent (Polyplus-transfection Inc., Illkirch, France) according to the manufacturer’s protocol. In 24 h, myoblasts were differentiated and experiments were started in another 96 h when myotubes were formed. Plasmids encoding constitutively active p38α and p38β isoforms 57 were transfected into C2C12 myoblasts using the jetPRIME reagent. Empty vector was transfected as the control. These manipulation of p38 MAPK in myoblasts did not alter the end result of differentiation and formation of myotubes 29.

### Real-time PCR

Total RNA was isolated from myotubes or muscle by using TRIzol reagent (Invitrogen, Carlsbad, CA). Real-time PCR was performed as described previously 21. Sequences of specific primers are listed in Table 1. Data were normalized to GAPDH.

**Table 1:** TABLE 1. PCR primers used in this study.

**Primer**	**Sequence**	
C/EBPβ forward	5′-GACAAGCTGAGCGACGAGTACA	
C/EBPβ reverse	5′-CGACAGCTGCTCCACCTTCTTC	
LC3b forward	5’-CGTCCTGGACAAGACCAAGT	
LC3b reverse	5’-ATTGCTGTCCCGAATGTCTC	
GABARAPL1 forward	5’-CATCGTGGAGAAGGCTCCTA	
GABARAPL1 reverse	5’-ATACAGCTGGCCCATGGTAG	
Atg12l forward	5’-GGCCTCGGAACAGTTGTTTA	
Atg12l reverse	5’-CAGCACCGAAATGTCTCTGA	
Beclin1 forward	5’-GGCCAATAAGATGGGTCTGA	
Beclin1 reverse	5’-CACTGCCTCCAGTGTCTTCA	
Atg4b forward	5’-ATTGCTGTGGGGTTTTTCTG	
Atg4b reverse	5’-AACCCCAGGATTTTCAGAGG	
GAPDH forward	5’-CATGGCCTTCCGTGTTCCTA	
GAPDH reverse	5’-GCGGCACGTCAGATCCA	**Product size**
LC3b -122 forward	5’-GACAGTTAACAGATGCTC	198 bp
LC3b -122 reverse	5’-AGTCCGCAGCCGAGTCAG	
LC3b -347 forward	5’-GAGCCACAAGATCAGATC	195 bp
LC3b -347 reverse	5’-TCATTCCCCTTCAGTCCT	
LC3b -711 forward	5’-TGATTGCTCACCAACCAA	198 bp
LC3b -711 reverse	5’-GACAACACCTGCTGTGCA	
Gabarapl1 -191 forward	5’-AACTACCCTACAGGTGAT	201 bp
Gabarapl1 -191 reverse	5’-CATCAAGGTTAGAAGAAG	
Gabarapl1 -398 forward	5’-CTTATAAAATGAATGGAT	199 bp
Gabarapl1 -398 reverse	5’-CTCCTTCTGACTGGTGTC	
Gabarapl1 -618 forward	5’-CATAAGGACACCAGTCAG	205 bp
Gabarapl1 -618 reverse	5’-GATGCCCAAAACAAACAC	
Gabarapl1 -829 forward	5’-CATTGCTCTGGAAACAGC	197 bp
Gabarapl1 -829 reverse	5’-CCAAGCTTTTCCTAAACC	

### Western blot analysis

Western blot analysis was carried out as described previously 24. Antibodies to total and/or phosphorylated p38MAPK (T181/Y182), p-C/EBPβ (T188), p-ULK1 (S555), total and phosphorylated AMPK (T172), p62 as well as p38α and p38β were from Cell Signaling Technology (Beverly, MA). Antibody to C/EBPβ and ULK1 were from Santa Cruz Biotechnology (Santa Cruz, CA). Antibody to atrogin1/MAFbx was from ECM Biosciences (Versailles, KY). Antibodies to UBR2 and LC3-II were obtained from Novus Biologicals (Littleton, CO). Anti-MHC antibody (MF-20) was from R&D Systems (Minneapolis, MN). Antibody to the HA tag was from Covance (Princeton, NJ, USA). Data was normalized to α-Tubulin (antibody was from Development Studies Hybridoma Bank at the University of Iowa, Iowa City, IA) or GAPDH (Antibody was from Millipore, Billerica, MA, USA). Levels of phosphorylated proteins were normalized to corresponding total proteins.

### Chromosome immunoprecipitation (ChIP) assay

ChIP assay was performed as previously described 21. Antibody against C/EBPβ was from Santa Cruz Biotechnology. Pre-immune IgG was from Sigma-Aldrich. The PCR primers used are listed in Table 1.

### Histology and confocal microscopy studies

Cross sections of TA were fixed and stained with H&E by the Histology Core at Lester and Sue Smith Breast Center, Baylor College of Medicine. Cross-sectional area of stained muscle sections was quantified by using the ImageJ software (NIH). Five view-fields with ~100 myofibers per field in each section were measured. Frozen sections of mouse TA (5 μm) that expressed GFP-LC3 and/or p38 MAPK mutant were stained with DAPI and examined with a Nikon A1R Confocal Laser Microscope using 60X objective.

Adjustment of brightness, contrast, color balance, and final image size was achieved using Adobe Photoshop CS (Adobe Systems, San Jose, CA, USA).

### Mapping of phosphorylated amino acid residues in ULK-1 

HEK293T cells (American Type Culture Collection, Manassas, VA, USA) cultured in 150 mm plates that were ~50% confluent were co-transfected with a plasmid encoding mouse ULK-1 with a FLAG tag (Addgene) and a plasmid encoding constitutively active p38β 58 (10 μg each) using the jetPRIME reagent (Polyplus-transfection Inc., Illkirch, France). The cell culture medium was replaced with fresh medium at 24 h. After an additional incubation of 24 h cells were lysed in RIPA buffer (50 mM Tris- HCl (pH 7.5), 150 mM NaCl, 2 mM EDTA, 1% NP-40, 0.1% SDS, 2 mM phenylmethylsulphonylfluoride (PMSF), 0.5% sodium deoxycholate, 1 mM NaF, 1/100 protease inhibitor cocktail, and 1/100 phosphatase inhibitor cocktail (Sigma-Aldrich, St. Louis, MO, USA). ULK-1 in cell lysates was precipitated using FLAG-M2 magnetic beads (Sigma-Aldrich) and subjected to 10% SDS-PAGE. The gel was then stained with Coommassie Blue R-250. The ULK1 band was cut out and subjected to tryptic phosphopeptide mapping conducted by Taplin Mass Spectrometry Facility at Harvard Medical School using an LTQ-Orbitrap mass spectrometer (Thermo Electron, West Palm Beach, FL, USA).

### Statistical analysis

Data are presented as the mean ± S.D. and were analyzed with one-way ANOVA or Student t test using the SigmaStat software (Systat Software, San Jose, CA) as indicated. When applicable, control samples from independent experiments were normalized to a value of 1 without showing variations (actual variations were within a normal range). Chi-square analysis was carried out by using R to compare the distributions of muscle fiber cross-sectional area among various groups. A p value < 0.05 was considered to be statistically significant.

## SUPPLEMENTAL MATERIAL

Click here for supplemental data file.

All supplemental data for this article are also available online at http://www.cell-stress.com/researcharticles/p38%ce%b2-mapk-mediates-ulk1-dependent-induction-of-autophagy-in-skeletal-muscle-of-tumor-bearing-mice/.

## Abbreviations

AMPK: AMP-activated Protein Kinase
C/EBPβ: CCAAT/enhancer-binding protein beta
GFP: Green fluorescence protein
HSP: Heat Shock Protein
IL-6: Interleukin 6
LCM: LLC Conditioned Medium
LLC: Lewis Lung Carcinoma
MAPK: Mitogen-Activated Protein Kinase
MKO: Muscle specific Knock Out
TA: Tibialis Anterior
TLR4: Toll-Like Receptor 4
ULK1: Unc-51 Like autophagy activating Kinase 1
UPP: Ubiquitin-Proteasome Pathway
