# Supplementary material for: p38β MAPK mediates ULK1-dependent induction of autophagy in skeletal muscle of tumor-bearing mice
Source: Cell Stress. 2018 Oct 10;2(11):311–24. doi: 10.15698/cst2018.11.163 (PMC6551802; doi:10.15698/cst2018.11.163)
Supplement: Supplementary file 1 [file ces-02-311-s01.pdf]

Reference	Fragment sequence	Site Position
sp|O70405|ULK1_MOUSE	K.DRMDFDEFFHHPFLDAS#TPIKK.S	281
sp|O70405|ULK1_MOUSE	K.TLTS#PADAAGFLQGSR.D	330
sp|O70405|ULK1_MOUSE	K.TLTSPADAAGFLQGS#R.D	341
sp|O70405|ULK1_MOUSE	R.YGAS#VPIPVPTQVHNYQR.I	429
sp|O70405|ULK1_MOUSE	R.VPS#PQGADVR.V	521
sp|O70405|ULK1_MOUSE	R.VGRS#PRPGSSVPEHSPR.T	532
sp|O70405|ULK1_MOUSE	R.SPRPGSSVPEHS#PR.T	543
sp|O70405|ULK1_MOUSE	R.LHS#APNLSDFHVVRPK.L	555
sp|O70405|ULK1_MOUSE	R.PLRGS#PKLPDFLQR.S	604
sp|O70405|ULK1_MOUSE	R.S#PLPPILGSPTK.A	614
sp|O70405|ULK1_MOUSE	R.QGVVMT#PPRNR.T	653
sp|O70405|ULK1_MOUSE	R.SFS#TSR.I	693
sp|O70405|ULK1_MOUSE	R.GGGASSPAPVVFTVGSPPS#GATPPQSTR.T	760
sp|O70405|ULK1_MOUSE	R.MFS#VGSSSSLGSTGSSSAR.H	774
sp|O70405|ULK1_MOUSE	K.GSASEAAGGPEYQLQESVVADQIS#QLSR.E	889
sp|O70405|ULK1_MOUSE	K.ASVVSCQGLS#LR.L	955
sp|O70405|ULK1_MOUSE	R.LILS#HAVQMVQSAALDEMFQHR.E	983
sp|O70405|ULK1_MOUSE	R.LILSHAVQMVQS#AALDEMFQHR.E	991
sp|O70405|ULK1_MOUSE	R.RLS#ALLSGVYA.	1043
sp|O70405|ULK1_MOUSE	R.LSALLS#GVYA.	1047

Table S1. Amino acid residues in overexpressed FLAG-ULK1 that are phosphorylated in the presence of overexpressed active p38β MAPK in HEK293 cells (see Material and Method for experimental procedure). # denotes phosphorylated residues.
